# Oncoplastic Nipple-Sparing Mastectomy and Immediate Reconstruction in Non-ideal Candidates

**DOI:** 10.7759/cureus.90157

**Published:** 2025-08-15

**Authors:** Aaron Dadzie, Yossef Alsabawi, Sonia Y Khan, Paul A Berry

**Affiliations:** 1 Medical Education, University of Texas Rio Grande Valley School of Medicine, Edinburg, USA; 2 General Surgery, University of Texas Rio Grande Valley School of Medicine, Edinburg, USA; 3 Plastic Surgery, DHR (Doctors Hospital at Renaissance) Health Plastic &amp; Reconstructive Surgery Institute, Edinburg, USA

**Keywords:** implant-based breast reconstruction, mastectomy, nipple-sparing mastectomy, oncoplastic breast surgery, plastic surgery

## Abstract

Mastectomies have long been used as a surgical treatment for malignant and benign disorders of the breast, both as a therapy and prophylaxis in high-risk patients. A nipple-sparing mastectomy (NSM) allows for the removal of most of the glandular and ductal tissues while preserving the surface architecture of the nipple-areola complex (NAC). This report summarizes the current mastectomy practices and suggests an expansion of the established surgical selection criteria for NSM in the context of immediate implant-based breast reconstruction. We present two cases in which a novel hybrid oncoplastic approach was used and discuss its safety and efficacy when performing primary breast reconstruction in female patients considered non-ideal candidates for NSM.

## Introduction

Nipple-sparing mastectomy

A mastectomy is the complete surgical removal of the tissue of the breast and is the only surgical option for breast cancer risk reduction [1]. One of the many modifications to this classic procedure is the nipple-sparing mastectomy (NSM). An NSM is a technique that allows for the removal of the major ducts from within the nipple lumen while leaving the dermis and epidermis of the nipple [2]. The NSM has already been proven to be a safe and efficacious oncological treatment for breast cancer and is regarded to have a superior aesthetic outcome compared to other mastectomy techniques [3,4]. The technical aspects of this procedure were studied by Moyer et al. [4].

Indications

Risk-reducing mastectomy is primarily indicated for those with documented genetic mutations such as BRCA1/2 mutations [5], and has both therapeutic and prophylactic functions.

While contraindications to NSM will vary across institutions and practitioners, there are no absolute contraindications. These include signs of nipple-areola complex (NAC) involvement, locally advanced breast cancer affecting the skin, inflammatory breast cancer, and bloody nipple discharge [6]. Additionally, factors such as significant ptosis, large breast size or high BMI, and active smoking are considered to be relative contraindications and, therefore, these patients are not considered optimal candidates for risk-reducing NSM [5]. Tumor size and tumor-to-nipple distance are also significant considerations when determining procedure eligibility.

The indications for NSM are evolving and broadening over time. Coopey et al. expanded the eligibility criteria for NSM, including patients formerly considered to have relative contraindications [6]. They argued that NSM can be safely performed on a tumor of any size so long as there is no evidence of NAC or skin involvement [6]. The previous exclusion criteria regarded tumor size greater than 3.0-3.5 cm as a contraindication for this procedure. Coopey et al. also argued including patients with a tumor-to-nipple distance greater than 1.0-2.5 cm, as long as the subareolar/nipple margin is pathologically negative [6].

Risks and Benefits

The NSM undoubtedly holds a superior aesthetic outcome than other mastectomy techniques. While some researchers, such as Ueda et al., reported no differences in the cosmetic outcomes between an NSM group and a skin-sparing mastectomy (SSM) group, nipple salvage at the time of mastectomy is largely considered the superior approach when it comes to aesthetics [4,7]. Patients' aesthetic satisfaction with their reconstruction plays a role in their quality of life and is highly dependent on the presentation of the nipple, such as its symmetry and shape [8]. This is a key factor for preferring NSM, as this technique allows for the salvage of the native nipple.

Nipple necrosis is a documented complication of NSM. Houvenaeghel et al. found complete and partial NAC necrosis in 3% and 15% (n=59) patients, respectively, undergoing prophylactic NSM [9]. The complication rate of NSM has been reported to be higher compared to that of SSM. In a systematic review, Agha et al. reported complication rates of 22.6% compared to 14%, respectively, for therapeutic NSM versus SSM [10]. The higher complication rate was attributed to the rate of nipple necrosis, total and partial, in the NSM group (15%) [11].

Oncoplastic breast surgery

Oncoplastic breast surgery encompasses a wide range of surgical techniques, all to resect a cancer while preserving the natural contour of the breast [12]. Oncoplastic surgical techniques also allow surgeons to address the resection of a neoplasm while still producing aesthetic outcomes.

The standard NSM or SSM performed on a patient with pre-existing unsatisfactory breast shape, according to their personal opinion, will still maintain that relative shape. Oncoplastic breast surgery allows patients’ aesthetic concerns to be addressed in the same procedure as resection.

Indications

While there are many reasons one may want to go with an oncologic approach, there are two characteristics that yield greater consideration [13]. The first is a large tumor burden relative to breast size. Poor cosmetic results occur when over 20% of the breast volume is removed using standard NSM and SSM approaches [14]. Thus, for patients where this is a necessity, an oncoplastic approach should be considered. The other factor is if oncologic resection requires repositioning of the NAC. This can often occur to address ptosis or to maintain the natural shape of the breast [12].

Implant-based breast reconstruction

Implant-based breast reconstruction is a technique that has existed for decades and has uses in both reconstructive and cosmetic applications. This report will focus on the reconstructive applications of this technique. The use of implants can be seen after a mastectomy, both for prophylaxis and breast cancer resection, to help restore the normal appearance of the breast [11]. Implants can be placed in the same procedure as the mastectomy or done at a later time, at times with the use of tissue expanders.

Herein, we present two cases of oncoplastic NSM with immediate reconstruction in non-ideal candidates. Informed written consent was obtained from both patients for the open-access publication of this case report.

## Case presentation

Patient 1

Patient 1 was a 47-year-old woman who had been referred to our clinic for a bilateral prophylactic mastectomy due to a strong family history of breast cancer. Family history was remarkable for leukemia (father), stomach cancer (mother), breast cancer (sister; BRCA status unknown), and another sister who tested BRCA positive and subsequently underwent a mastectomy. The patient had elected not to undergo BRCA screening. The patient expressed concern over her family history and interest in undergoing a mastectomy with reconstruction. A routine mammogram five months prior to presentation showed heterogeneously dense breasts with no evidence of malignancy. Subsequent breast MRI was also unremarkable. Based on the Tyrer-Cuzick model, the patient had a 30% chance of developing breast cancer. She expressed her preference to stay around her current size (a D-cup) post-reconstruction.

**Figure 1:**
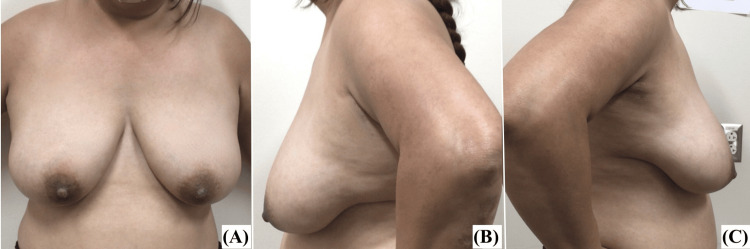
Patient 1, preoperative images: (A) AP view, (B) left lateral view, (C) right lateral view

She had a BMI of 32 kg/m² and was a non-smoker. Pre- and postoperative characteristics of the patient's breasts are detailed in Table 1, while preoperative photographs are presented in Figure 1.

**Table 1:** Breast characteristics before and after staged reconstruction of both breasts (Patient 1) SNN: sternal notch to nipple

	Preoperative		Postoperative	
Breast	Right	Left	Right	Left
Size (g)	~750	~700	~750	~750
Ptosis grade	2	2	1	1
SNN distance (cm)	30.5	31.0	24.0	24.0

The patient underwent a bilateral NSM with concurrent reconstructive lift four months later. She was followed up at monthly intervals to track the progression of healing and for the evaluation of bedside expansion. At her five-month follow-up, she was noted to have healed well and was ready to proceed with the next phase of her reconstruction: exchange of bilateral retained tissue expanders with breast prostheses.

**Figure 2:**
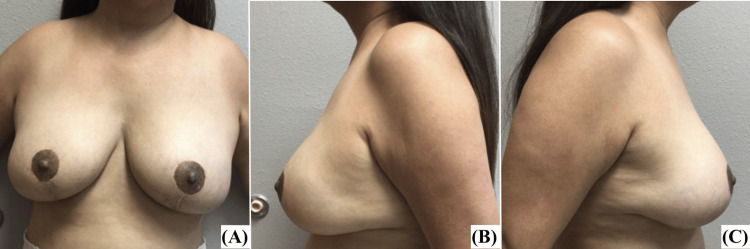
Patient 1 five months after staged reconstruction of left and right breasts by exchange of tissue expanders for a permanent prosthesis: (A) AP view, (B) left lateral view, (C) right lateral view

At six months, the patient underwent staged reconstruction of the left and right breasts by exchange of tissue expanders for a permanent prosthesis. The nipples were transposed to 24 cm from the suprasternal notch. She was seen for follow-up at three and six months, and her recovery was within normal limits. Postoperative photographs are presented in Figure 2.

Of note, at the six-month follow-up, the patient raised concerns of a persistent 9 x 6 x 6 mm nodule in the left NAC at the 9 o’clock position that was later confirmed to be fat necrosis on pathology. To treat the persistent left-sided nodule and address her concerns regarding her breast contour, she underwent excision of the left breast mass with revision mastopexy. The patient has maintained follow-up at three-month intervals and has progressed well.

Patient 2

Patient 2 was a 41-year-old woman presenting to our clinic with concerns regarding the potential of a poor aesthetic outcome after the surgical resection of her cancer. Approximately six weeks prior to her presentation, she was diagnosed with malignancy of the left breast. At that time, the patient underwent imaging investigations, including ultrasound, MRI, and mammography, that confirmed the presence of a solid irregular mass measuring 1.0 x 1.7 cm. The diagnostic mammogram revealed the presence of malignant-appearing calcification in the lower outer quadrant of the breast. A biopsy of the mass indicated an estrogen receptor positive, progesterone receptor positive, herceptin 2 negative, Nottingham grade 2 invasive carcinoma with mucinous features in the left breast. The mass was measured at 4 mm in its greatest dimension and was reported as suspicious for lymphovascular invasion. The biopsy was also significant for adjacent intermediate- to high-grade ductal carcinoma in situ with comedo necrosis and intraductal microcalcifications. Her plan was to undergo extirpative therapy via bilateral NSM. Her oncology team deemed adjuvant chemotherapy or radiation therapy unnecessary for optimal treatment.

**Figure 3:**
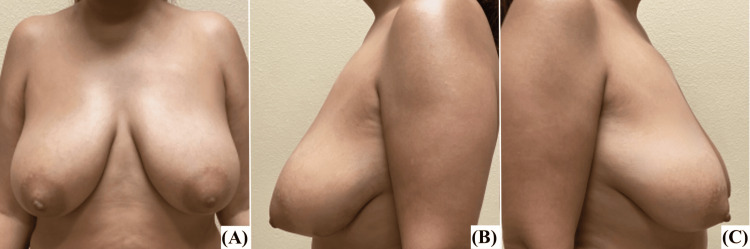
Patient 2, preoperative images: (A) AP view, (B) left lateral view, (C) right lateral view

She presented with bilateral grade 2 breast ptosis. She had a BMI of 30 kg/m² and was a non-smoker. Pre- and postoperative characteristics of the patient's breasts are detailed in Table 2, while preoperative photographs are presented in Figure 3.

**Table 2:** Breast characteristics before and after staged reconstruction of both breasts (Patient 2) SNN: sternal notch to nipple

	Preoperative		Postoperative	
Breast	Right	Left	Right	Left
Size (g)	~1100	~1100	~800	~800
Ptosis grade	2	2	1	1
SNN distance (cm)	30.5	31.0	24.0	24.0

One week after presentation, the patient underwent right-sided NSM with bilateral immediate reconstruction by modified mastopexy and placement of tissue expanders. The left-sided mastectomy was performed by a general surgeon prior to this procedure.

**Figure 4:**
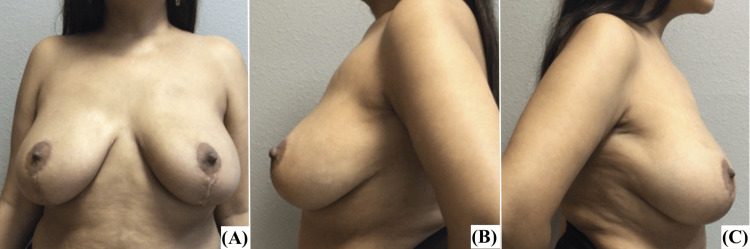
Patient 2 four months after staged reconstruction of left and right breasts by exchange of tissue expanders for a permanent prosthesis: (a) AP view, (b) left lateral view, (c) right lateral view

Three months later, the patient underwent staged reconstruction of the acquired right and left breast deformity by exchange of tissue expanders for permanent prosthesis and operative treatment of right chest wall wounds/scars, and is now healing within normal limits. The nipples were transposed to 24 cm from the suprasternal notch. At the four-month follow-up, the patient was recovering within normal limits and had grade 1 ptosis bilaterally. Postoperative photographs are presented in Figure 4.

## Discussion

Traditional breast reconstruction techniques using expanders would normally require an additional operation beyond the mastectomy and reconstruction that would either reposition or reconstruct the nipple. As reported here, we performed NSM on women who, under traditional guidelines, would not be ideal candidates for NSM. This allowed us to achieve an aesthetically pleasing, repositioned nipple-areola complex at the time of extirpative operation. Despite a greater degree of ptosis, the nipple could be moved, and the contour improved at the time of NSM. Our patients' greater degree of mammoptosis would preclude them from standard considerations. We have determined that NSM is a viable option for these women for optimal aesthetic results while additionally sparing them from the risks associated with additional operations for nipple reconstruction or repositioning, lowering the healthcare burden on them as well as the healthcare system.

There are several key considerations when determining whether a patient is an appropriate candidate for this course of action. In the case of these two patients, they were not particularly high risk in terms of flap loss. They were relatively healthy women, with BMIs under 35, and non-smokers. High-BMI women tend to have poor blood flow to the flaps due to excess fatty tissue and ptosis, meaning that, at baseline, the flap is more likely to fail [15]. Smoking also imparts a heavy toll on attempts to keep a flap alive, and it would likely be disastrous to attempt this technique on a smoker [15]. Another consideration is how far the nipple is being moved. In the case of our two patients, the nipples were transposed to 24 cm from the suprasternal notch. If a desirable result requires moving the nipple over larger distances, this approach may not be appropriate due to a larger surface area that must be perfused, further distances for the perforates to travel, greater intraoperative traction of the flaps, and more manipulation of the NAC during reconstruction [15].

The last major consideration is patient satisfaction with the reconstruction. Beyond the patients' statements that they are satisfied with the reconstruction, we have no quantitative data on their satisfaction. However, this is an area that presents opportunities for future study. Using a validated quality of life and patient satisfaction tool, such as BREAST-Q, as part of a prospective study could help determine if a new standard of care should be adopted for patients who meet these criteria [16].

The current evidence indicates that NSM with immediate implant-based breast reconstruction is oncologically safe in appropriately selected patients and is associated with low rates of major complications and nipple necrosis. Large retrospective series report major complication rates around 9% and nipple necrosis rates near 1%, with higher risks observed in patients with preoperative radiotherapy, active smoking, and periareolar incisions. Direct-to-implant reconstruction is associated with lower rates of nipple necrosis and explantation compared to tissue expander-based approaches. The use of acellular dermal matrix or mesh does not appear to increase complication rates [17].

However, patient-reported outcomes are mixed. Some studies show that NSM provides higher satisfaction with breast appearance and sexual well-being compared to SSM, while others find no significant difference in satisfaction or quality of life between NSM and SSM when matched for clinical factors [18-20]. Complication rates for NSM and SSM are generally similar, but recent data suggest that NSM may be associated with a higher risk of surgical site infection and wound complications requiring surgical intervention, even when periareolar incisions are uncommon [21].

Technical refinements, such as the choice of incision and intraoperative perfusion assessment, have led to a reduction in complication rates over time. NSM with immediate implant-based reconstruction is feasible using both subpectoral and prepectoral approaches and can be performed safely via conventional or minimally invasive techniques (e.g., endoscopic or single axillary incision) in well-selected patients, with comparable oncologic outcomes [17,22-24].

In summary, NSM with immediate implant-based reconstruction is a well-established option, characterized by favorable safety and patient satisfaction profiles; however, patient selection and surgical technique are crucial in minimizing complications [17,18,20-22,24].

## Conclusions

NSM has emerged as an increasingly popular option for both cancer treatment and prophylaxis, owing to its significant reconstructive benefits. This report adds to the growing body of literature by highlighting the expanding indications for NSM, particularly in patients who were once considered non-ideal candidates for the procedure. As the criteria for patient selection evolve, it is essential for future studies to further explore clinical outcomes and patient satisfaction to better understand the long-term implications of NSM. This will help refine surgical guidelines and optimize the balance between oncological safety and aesthetic restoration, ensuring the best possible outcomes for a broader range of patients.
